# A case of testicular leiomyoma in androgen insensitivity syndrome: exploring malignancy controversies

**DOI:** 10.1093/omcr/omae170

**Published:** 2025-01-18

**Authors:** Neha Neupane, Devish Pyakurel, Abha Shrestha, Keyoor Gautam, Srijana Shrestha, Santosh Pradhan, Vivek Pant

**Affiliations:** Department of Pathology, Samyak Diagnostic Pvt Ltd, Yala Sadak, Kathmandu 44600, Nepal; Department of Pathology, Samyak Diagnostic Pvt Ltd, Yala Sadak, Kathmandu 44600, Nepal; Department of Pathology, Samyak Diagnostic Pvt Ltd, Yala Sadak, Kathmandu 44600, Nepal; Department of Pathology, Samyak Diagnostic Pvt Ltd, Yala Sadak, Kathmandu 44600, Nepal; Department of Pathology, Kantipur Dental College, Basundhara Road, Kathmandu 44600, Nepal; Department of Biochemistry, Samyak Diagnostic Pvt Ltd, Yala Sadak, Kathmandu 44600, Nepal; Department of Biochemistry, Samyak Diagnostic Pvt Ltd, Yala Sadak, Kathmandu 44600, Nepal

**Keywords:** androgen insensitivity syndrome (AIS), leiomyoma, testis, smooth muscle actin (SMA), Desmin

## Abstract

Testicular leiomyoma is an exceptionally rare finding in patients with androgen insensitivity syndrome (AIS). Here, we present a report of a 30-year-old individual diagnosed with complete AIS who presented with an inguinal mass subsequently identified as a right sided testicular leiomyoma. While leiomyoma are generally considered benign, controversies persist regarding the potential for malignancy in inguinal masses among AIS patients. This case underscores the complexity of diagnosing and managing such rare occurrences, particularly when considering the diverse opinions found in the scientific literature regarding the malignant potential of inguinal masses in AIS individuals. By presenting this case, we contribute to the ongoing debate on the evaluation and treatment of inguinal masses in AIS patients, emphasizing the need for further research and consensus in this area.

## Introduction

Androgen insensitivity syndrome (AIS) is an X-linked disorder where an affected individual is phenotypically female having 46 XY karyotype and with normal differentiation of the testicles, with no uterus. It may manifest during puberty with primary amenorrhea or during early life as an inguinal tumor [1]. The undescended gonads are prone to the development of several types of tumors presenting as inguinal mass.

Reproductive organ development occurs between 8 to 14 weeks of gestation and is influenced by the presence of androgens and functional androgen receptors (AR). The AR gene, located on the Xq11-12 region, contains three ligand-dependent transcription factors responsible for its major functions. In AIS, abnormal androgen secretion or resistance at the receptor level leads to a spectrum of phenotypic presentations. The severity of AIS depends on the residual functionality of the AR, and manifestations can vary due to defective testosterone binding. As a result, individuals with AIS may present with cryptorchidism instead of typical female gonads, and these undescended testes are at higher risk for developing tumors, including testicular leiomyomas. Recent studies have suggested that the abnormal hormonal environment and impaired AR signaling in AIS contribute to the development of these tumors [2].

Among the various inguinal tumors, leiomyomas are exceptionally rare and are typically diagnosed only following surgical excision. Leiomyomas are benign mesenchymal tumors originating from smooth muscle cells. While they most commonly arise in the uterus, small intestine, and esophagus, they have also been reported in the renal pelvis, urinary bladder, prostate, epididymis, scrotum, and glans penis [3]. AIS, previously termed testicular feminization syndrome, is a rare condition with a prevalence ranging from 1 in 204 000 to 1 in 991 000 in genetic males [4]. The risk of gonadal tumors in AIS patients’ increases with age, with an incidence reported between 0.8% and 22% in individuals retaining gonads into adulthood [5].

If AIS is suspected, laboratory workup for diagnosis generally starts with sex chromosomes analysis to determine if genetic sex differs from physical appearance. Thereafter, sex hormone levels are measured typically revealing high testosterone. An ultrasound can confirm the absence of internal female reproductive organs. Children with AIS often have female external genitalia but lack a womb and ovaries. During hernia repair, if testicles are suspected in the abdomen, a biopsy may be taken to confirm the presence of testicles instead of ovaries. These diagnostic steps help confirm AIS. Given the increased risk of malignant transformation in undescended testes, early gonadectomy is recommended.

However, there have been mixed reports on malignancy potentials of these undescended testes in cases of AIS. The risk of malignancy is estimated to be 3.6% at 25 years and 33% at 50 years if testes remain in abdomen in patient with complete AIS [6]. The mixed reports are attributed to the reporting of very few cases of testicular leiomyoma. Albert and miniberg reported the first case of testicular leiomyoma in 1972 which was located in the tunica albuginea [7]. A literature search conducted on PubMed in June 2011 identified a total of 17 reported cases [8]. Additionally, over the past 10 years, only 32 cases have been published, further highlighting the rarity of this condition. The recommended guideline for cryptoorchidism correction is before 2 years [9]. A prevailing hypothesis suggests that the mechanism leading to testicular cancer may be established during fetal development, with the associated risk largely determined during the intrauterine period or early childhood [10, 11]. If this theory is accurate, the timing of surgical intervention, such as orchiopexy, would not substantially affect the incidence of testicular cancer. While some studies have indicated that early orchiopexy may reduce the risk of testicular cancer, the available data remain inconclusive [12, 13]. We report a case of 30 years patient, previously diagnosed with AIS, having intratesticular leiomyoma who was provisionally diagnosed as having gonadoblastoma.

## Case presentation

A 30-year-old female presented to the emergency department with complaints of swelling, right-sided groin pain, and a sensation of heaviness while walking and lifting heavy objects. The patient was from a remote region in Nepal. Despite being diagnosed with AIS before puberty, she had not pursued further medical care due to social stigma and peer pressure. Unfortunately, this situation underscores the challenges faced by patients in similar circumstances. She had been amenorrheic throughout her life and though was previously diagnosed with testicular feminization syndrome and inguinal hernia but did not seek treatment for these conditions. She denied history of trauma and symptoms of genitourinary infection. She had no history of other major illness and was not under any medication. On physical examination she had normal breast development and external genitalia appeared normal but her axillary and pubic hair was sparse. Chromosomal analysis revealed a male karyotype, indicative of a disorder of sex development. Cytogenetic analysis identified a balanced translocation between the long arms of chromosome 3 at the q29 region and chromosome 11 at the q21 region in all metaphases studied. Additionally, tumor markers including alpha-fetoprotein (AFP), beta-human chorionic gonadotropin (B-HCG), and CA125 were measured, all of which were found to be within normal ranges. The ultrasonography (USG) report revealed that uterus and bilateral adnexal structures were absent. Right gonad along with gonadal vessels was visible till level of mid-inguinal region. Left gonad along with gonadal vessels was at the level of deep inguinal region.

**Figure 1:**
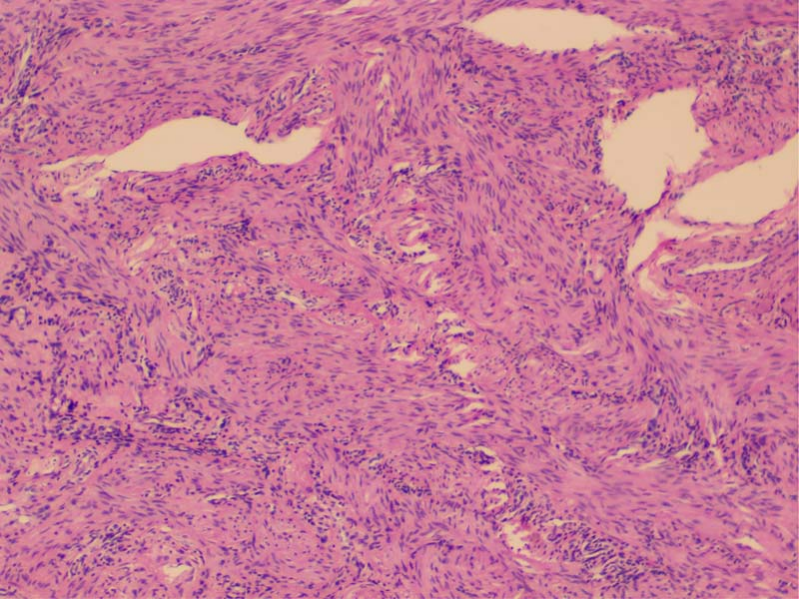
Tumor cells arranged in interlacing fascicles and bundle. Individual cells exhibited blunt elongated nuclei, few with tapered ends and eosinophilic cytoplasm.

**Figure 2:**
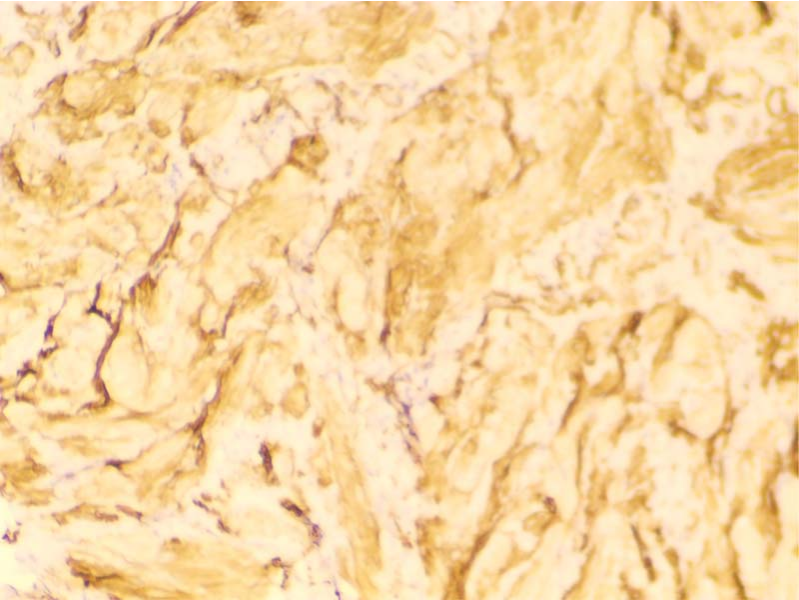
Smooth muscle actin (SMA) positive tumor cells.

**Figure 3:**
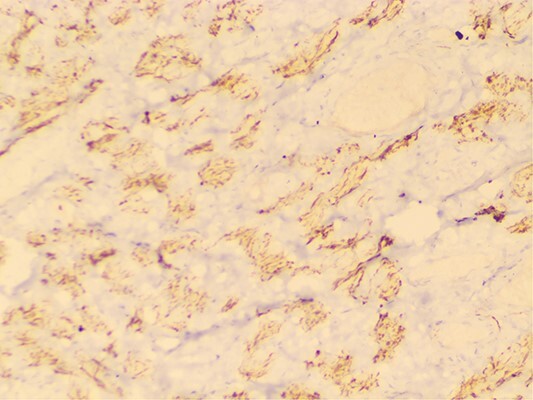
Desmin positivity in tumor cells.

The attending surgeon made the provisional diagnosis of right sided malignant testicular mass owing to the patients past history and radiological findings. Laparoscopic bilateral gonadectomy was performed and at the time of surgery, right gonad was firm on palpation and cut- section revealed small solid component. With the provisional diagnosis of right sided gonadoblastoma, tissue sample was sent to our centre for further evaluation. Gross pathological examination revealed two pieces of fibrocystic tissue, measuring 5 × 4 cm and 5 × 3 cm respectively and cut section revealed clear fluid. Slides were prepared and carefully examined under hematoxylin and eosin stain. Microscopic examination revealed tumor cells arranged in interlacing fascicles and bundles (Fig. 1). Individual cells exhibited blunt elongated nuclei, few with tapered ends and eosinophilic cytoplasm. Adjoining areas comprised of areas of seminiferous tubules with normal morphology along with few inflammatory cells and focal areas of hemorrhages. Mitosis, necrosis, primitive germ cells, primitive sex cord stromal cells and ovarian stroma were not seen in the sections examined. Immunohistochemistry for smooth muscle actin (Fig. 2) and desmin (Fig. 3) were positive in the spindle cells and S100 was negative. Hence, the final diagnosis of “Leiomyoma of Testis” was made and reported to the operating surgeon. The patient’s postoperative follow-up has been favorable, with no recurrence of the disease six months after surgery.

## Discussion

Diagnosis of leiomyoma can be established through USG, magnetic resonance imaging (MRI), and computed tomography (CT) scans. USG is the preferred modality for initial evaluation of testicular pathology due to its wide availability, high sensitivity, and cost-effectiveness. MRI may be used to further characterize a testicular mass. However, only histopathological examination can confirm the diagnosis, with immunohistochemistry needed to determine the nature of spindle cells. Despite the diagnostic capabilities of imaging, surgery remains the treatment of choice due to the limitations of USG in distinguishing benign from malignant tumors [14, 15].

Immunohistochemical examination plays a crucial role in distinguishing testicular leiomyoma from other tumors. Specific markers such as SMA and desmin are utilized for this purpose. SMA is a smooth muscle-specific isoform of actin that is absent in striated muscle, making it a valuable marker for identifying smooth muscle tumors. Desmin, on the other hand, is a universal marker for muscle cells and is expressed in both smooth and striated muscle cells, further aiding in the classification of tumors. Regarding associated conditions with AIS, we conducted tumor marker evaluations, including AFP, B-HCG, and CA-125, which were performed in our case. Additionally, genetic testing was conducted through chromosomal analysis, revealing a male karyotype suggestive of disorders of sex development.

The treatment of AIS may involve the surgical removal of testes, either before or after puberty, to reduce the risk of testicular malignancy. This is typically accompanied by estrogen replacement therapy. Additional management includes vaginal dilation to prevent dyspareunia. To promote bone health, regular weight-bearing exercises, along with calcium and vitamin D supplementation, are recommended. Monitoring bone mineral density (BMD) is essential, and bisphosphonate therapy may be considered in cases of reduced BMD [16]. Evaluation of at-risk relatives should be conducted through karyotyping, molecular genetic testing for known AR variants, and androgen-binding assays for individuals where the AR variant is unknown. Psychological support and genetic counseling are crucial for both the patient and their family to help them understand the condition.

Despite the mixed views in scientific literature regarding the malignant potential of such masses, the decision for gonadectomy in our patient was made cautiously, considering both the rarity of the leiomyoma and the need to mitigate potential risks associated with malignancy.

## Conclusion

In conclusion, the case of our 30-year-old patient with AIS who presented with a testicular leiomyoma underscores the challenges in managing rare manifestations of this syndrome, particularly concerning the potential malignancy of inguinal masses.
